# Scanning Micro X-ray Fluorescence and Multispectral Imaging Fusion: A Case Study on Postage Stamps

**DOI:** 10.3390/jimaging10040095

**Published:** 2024-04-22

**Authors:** Theofanis Gerodimos, Ioanna Vasiliki Patakiouta, Vassilis M. Papadakis, Dimitrios Exarchos, Anastasios Asvestas, Georgios Kenanakis, Theodore E. Matikas, Dimitrios F. Anagnostopoulos

**Affiliations:** 1 Department of Materials Science and Engineering, University of Ioannina, 45110 Ioannina, Greece; fgerodim@uoi.gr (T.G.); i.patakiouta@uoi.gr (I.V.P.); d.exarchos@uoi.gr (D.E.); a.asvestas@uoi.gr (A.A.); matikas@uoi.gr (T.E.M.); 2 Department of Industrial Design and Production Engineering, University of West Attica, 12244 Athens, Greece; v.papadakis@uniwa.gr; 3 Institute of Electronic Structure and Laser, Foundation for Research and Technology, 70013 Hellas, Greece; gkenanak@iesl.forth.gr

**Keywords:** scanning micro-XRF, multispectral imaging, spectral images, dataset fusion, co-registration, k-means clustering, elemental and chemical analysis, postage stamps, material characterization

## Abstract

Scanning micrο X-ray fluorescence (μ-XRF) and multispectral imaging (MSI) were applied to study philately stamps, selected for their small size and intricate structures. The μ-XRF measurements were accomplished using the M6 Jetstream Bruker scanner under optimized conditions for spatial resolution, while the MSI measurements were performed employing the XpeCAM-X02 camera. The datasets were acquired asynchronously. Elemental distribution maps can be extracted from the μ-XRF dataset, while chemical distribution maps can be obtained from the analysis of the multispectral dataset. The objective of the present work is the fusion of the datasets from the two spectral imaging modalities. An algorithmic co-registration of the two datasets is applied as a first step, aiming to align the multispectral and μ-XRF images and to adapt to the pixel sizes, as small as a few tens of micrometers. The dataset fusion is accomplished by applying k-means clustering of the multispectral dataset, attributing a representative spectrum to each pixel, and defining the multispectral clusters. Subsequently, the μ-XRF dataset within a specific multispectral cluster is analyzed by evaluating the mean XRF spectrum and performing k-means sub-clustering of the μ-XRF dataset, allowing the differentiation of areas with variable elemental composition within the multispectral cluster. The data fusion approach proves its validity and strength in the context of philately stamps. We demonstrate that the fusion of two spectral imaging modalities enhances their analytical capabilities significantly. The spectral analysis of pixels within clusters can provide more information than analyzing the same pixels as part of the entire dataset.

## 1. Introduction

Scanning micro X-ray fluorescence (μ-XRF) is a noninvasive XRF imaging technique that allows the nondestructive visualization of the spatial distribution of elements in a macroscopic sample [1,2,3,4,5]. This is achieved by scanning the sample’s surface with an ionizing X-ray beam and analyzing the emitted fluorescence radiation. Multispectral imaging (MSI) is a noninvasive technique that captures images of materials at different wavelengths in contiguous narrow spectral bands, typically in the visible (Vis, 400−750 nm) and the near-infrared (NIR, 750−2500 nm) regions [6,7,8,9,10]. This imaging technique provides valuable spectral information for the object under investigation, allowing for material identification and spatial mapping. XRF and MSI are complementary techniques in various fields, including archaeology and art conservation, enabling detailed elemental and chemical analysis [11,12,13]. The produced information is crucial for identifying unknown materials and pigments and authenticating artworks or artifacts. For this reason, the correlation of the collected data by both techniques is essential for obtaining a comprehensive and holistic view of the objects under investigation. By combining the advantages of each mentioned modality, researchers and art conservators can achieve a more accurate, precise, and insightful analysis method [14,15,16,17].

Two distinct approaches characterize the trend toward multimodal analysis. The first approach involves using multiple techniques within a unified system to generate inherently co-registered and synchronized data cubes [16,17]. The second approach involves using distinct systems for asynchronous imaging measurements and the subsequent data co-registration and data fusion [14,15]. The asynchronous approach may be more cost-effective and less complex to implement but introduces challenges in ensuring accurate co-registration.

The present study analyzed complex-in-structure stamps using scanning μ-XRF and MSI imaging spectroscopies, with a pixel size of a few tens of micrometers in both directions. The individual datasets were acquired asynchronously. This work aims to analyze fused datasets from the two different spectral imaging techniques. This process involves two steps. Firstly, the datasets are co-registered by aligning the MSI and μ-XRF images and binning the smaller MSI pixel size to match the μ-XRF pixel size. For that purpose, a computer vision algorithm was applied to correlate the spectral hypercubes. Analogous methods have been employed for pigment investigation with spatial resolutions typically in millimeters [14]. Secondly, k-means clustering and sub-clustering of the multispectral and μ-XRF datasets were applied to extract information and draw conclusions about the studied stamps. Our study critically examines the merits and limitations of this refined methodological approach. The dataset fusion considerably enhances the analytical capability of the two spectroscopic imaging techniques. The proposed method can be used to combine datasets obtained through various asynchronous imaging techniques applied sequentially.

## 2. Instruments and Technical Characteristics

### 2.1. The μ-XRF Scanner

The μ-XRF scanning was realized with the M6 Jetstream (Bruker) scanner [2,18], which allows scan areas of 80 × 60 cm^2^. The M6 Jetstream is equipped with a 30 W rhodium X-ray tube. In the present measurement, the X-ray tube was operated at a high voltage of 50 kV and a current of 600 μA, while no absorption filter was applied on the beam path of the ionization radiation. The measurement was conducted by applying helium flux to enhance the detection of low-energy transitions from light elements such as Al and Si. The incoming X-ray beam from the source was focused using a polycapillary glass optic and impinged perpendicularly on the target surface. The detector detected photons emerging at an angle of about 60° relative to the target surface. A silicon drift detector of 30 mm^2^ active area was used for the photon detection, with an energy resolution of 138 eV at the Mn Kα-energy. Each spectrum consisted of 4096 channels.

**Figure 1:**
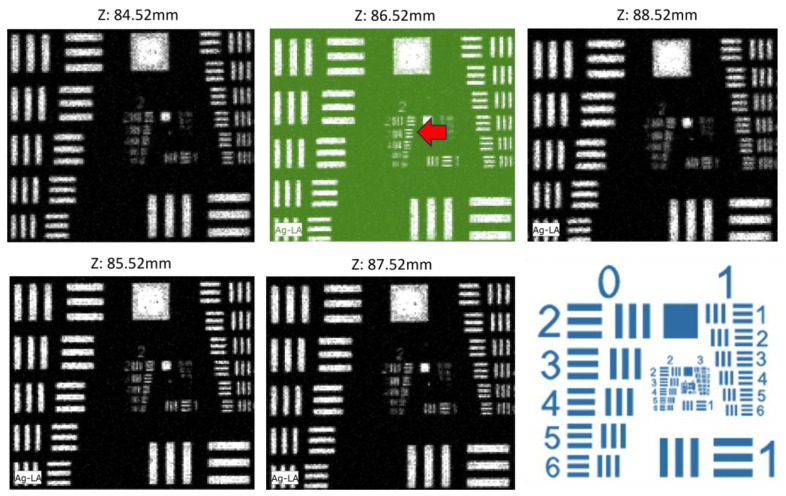
Ag Lα transition maps for the silver-printed USAF-1951 resolution chart (see text) as a function of the distance between the spectrometer head and target. The distance changes in a step of 1 mm. The image at the position z equal to 86.52 mm presents the best spatial resolution. The lines in Group 2, Element 3 (red arrow) of 100 μm width (Equation (1)) are resolved. (Bottom right) The USAF 1951 map.

In this study, achieving maximum spatial resolution was essential due to the complex spatial structures that stamps can present. For this reason, the distance of the measuring head from the target (stamp) was adjusted to achieve the highest spatial resolution. Initially, the measuring head was positioned at the focal point of the M6 Jetstream camera on the target (nominal beam spot of 100 μm for the Mo Kα). Subsequently, the spectrometer head’s position was fine-tuned to achieve the highest spatial resolution. This was done by changing the distance between the spectrometer head and a reference target in steps of a millimeter and scanning the target in each position. The reference target was a USAF 1951 resolution test chart [3,19,20,21]. The chart was printed in the lab using the CeraPrinter F-Series by Ceradrop^®^, depositing AGFA PRELECT SPS201 nano-silver ink onto Powercoat HD coated paper. This digital materials deposition platform seamlessly integrates inkjet and Aerosol Jet^®^ technologies, offering high precision with up to 15 μm resolution [22]. Each group in the USAF 1951 chart comprises six elements, each consisting of two blocks, each containing three parallel lines (vertical and horizontal, Figure 1). Based on the group number and element number, the width of a line in micrometers is given by [23]:

(1) $$\mathrm{width}\left(\mu m\right)=1000\cdot 2^{-\;\left(group\;+\;\frac{element\;+\;5}{6}\right)\;}$$

The sensitivity of the spatial resolution as a function of the spectrometer head position in steps of one millimeter is shown for the Ag Lα transition map in Figure 1. The image at the position z equal to 86.52 mm presents the best spatial resolution. The lines in Group 2, Element 3 of 100 μm width are resolved. The Ag Lα (2.7 keV) transition was chosen as polycapillary optics have lower spatial resolution at the low photon energy. Therefore, the spatial resolution measured improves further for higher transition energies.

### 2.2. The MSI Camera

The multispectral measurement was realized using the multispectral imaging system (XpeCAM X02, XpectralTEK, Braga, Portugal) [24]. The XpeCAM solution is composed of three major components. A broad wavelength illumination source, “LAMPA”, was employed, covering the wavelength range between 360 nm and 1200 nm, with a controllable selection of the illumination bands (UV, VIS/NIR, NIR/VIS). The multispectral imaging system incorporates a very sensitive 6.5 Mpixel sensor and a filter wheel that can hold 30 band-pass filters between 350 nm and 1200 nm. The system calibration and data acquisition processes are fully automated, with an autofocus mechanism that corrects the optical systems’ chromatic aberrations. At the same time, the equipment is portable and has a user-friendly interface. Following the acquisition, the acquired data can be uploaded to XpeCAM Cloud, a web application coupled with artificial intelligence technology incorporated into a cloud infrastructure. This combination enables automated processing, analytics, visualization, and reporting in a short time frame [25]. This complete process culminates in the formation of a high-quality MSI cube.

## 3. Applied Methodology and Results

### 3.1. The 0.25 Franc French Gallic Cock Stamp by Albert Decaris

**Figure 2:**
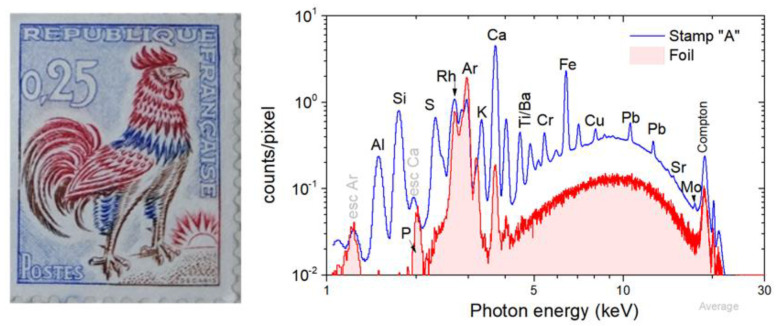
(**Left**) Stamp-A: 0.25 franc French Gallic cock stamp by Albert Decaris [26]. (**Right**) The mean XRF spectrum of stamp-A (sum spectrum divided by the number of the measured pixels) is compared with the mean XRF background spectrum (see text).

The investigated stamp, defined as stamp-A, is the 0.25 franc French Gallic cock stamp “Gallus gallus domesticus”, painted by Albert Decaris. Stamp-A is shown in Figure 2. The stamp, printed with dimensions of 20 × 26 mm², was issued for the first time in 1962 [26]. The primary criterion for selecting this stamp is its complex spatial distribution of pigments, which displays significant spatial variation in the order of a tenth of a millimeter. Furthermore, the presence of ultramarine pigment implies the existence of elements with low atomic numbers, like Al and Si [26]. Imaging of low-Z elements using XRF is challenging. The 0.25 franc French Gallic cock was analyzed through imaging X-ray fluorescence spectroscopy using the handheld Tracer 5i (Bruker) spectrometer with a 0.5 mm bore collimator by Shugar [27]. In the present study, the spatial resolution is nearly an order of magnitude higher depending on the recorded transition energy.

### 3.2. The Scanning μ-XRF Measurements

For the scanning μ-XRF measurement, the stamp was supported on a SpectroMembrane^®^ thin Mylar^®^ foil of 3.6 µm in thickness and 76.2 mm in diameter by Chemplex^®^ [28]. The M6 Jetstream was set at a top-down configuration. Helium flow was applied during all scans. It is worth mentioning that using helium flow during measurement significantly improves the detection limits of the M6 Jetstream, enabling the detection of elements with low atomic numbers, like Al and Si. Helium flow increased the detection of Al and Si Kα intensities by 15 and 5 times, respectively, during the measurement of pure aluminum and silicon targets. The pixel size for all scans was 50 × 50 μm^2,^ and the dwell time was 20 ms per pixel.

For the background determination, the supporting foil was measured in 1692 pixels (36 × 47). The background mean spectrum (defined as the sum spectrum divided by the number of pixels) is shown in Figure 2. On the spectrum, the scattered radiation of Rh K and L and Ar K transitions originate from the presence of argon in the atmosphere. In contrast, the presence of Ca and P, attributed to traces within the Mylar, is observed [28].

The scanned area of stamp-A was 20.2 × 31.8 mm^2^, and the measurement time was about 55 min. A total of 172,312 spectra were collected. The corresponding mean spectrum of the stamp’s scan is shown in Figure 2. The dominant observed transitions in the sample are the X-ray fluorescence transitions of Al, Si, S, K, Ca, Ti, Ba, Cr, Fe, and Pb and the faint Sr and Mo K X-ray transitions.

**Figure 3:**
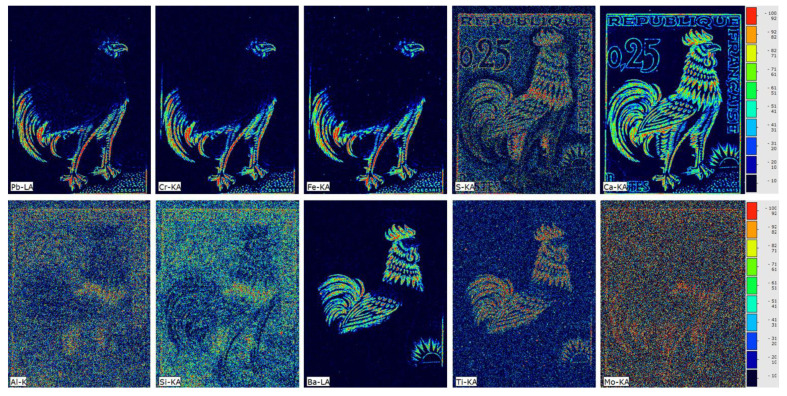
Elemental distribution maps were obtained through μ-XRF dataset analysis. (**Upper** from **left** to **right**) Pb Lα, Cr Kα, Fe Κα, S Κα, and Ca Κα. (**Lower** from **left** to **right**) Al Κα, Si Κα, Ba Lα, Ti Κα, and Mo Κα. A total of 172,312 pixels were acquired, each pixel having a size of 50 × 50 μm^2^.

The elemental transition maps acquired during the μ-XRF scanning are shown in Figure 3. The elemental distribution maps were generated via the built-in M6 Jetstream software (Esprit-M6 v.1.6). The Ca and S Kα intensity distributions are highly correlated in all the stamp regions containing pigments, indicating the use of calcium sulfate as an ingredient and/or extender for prime pigment substances. Furthermore, there is a strong correlation between the intensities of the Fe Kα, Cr Κα, and Pb Lα, indicating the presence of lead chromate (PbCrO_4_) with an iron-based ochre pigment in the brown-colored regions [27]. The correlation between Al and Si Κα transition maps confidently reveals the presence of ultramarine in the blue regions [27]. The Ba Lα elemental map corresponds to the red areas of the stamp. The Ti Kα elemental map exhibits a dispersed distribution on its surface, with higher concentration in areas where Ba is abundant. Given the energy closeness between the Ti Kα transition (4.51 keV) and the Ba Lα transitions (4.47 keV), special attention is required in interpreting elemental maps. The analysis presented below helps to distinguish the contribution of each individual element.

### 3.3. The MSI Measurements

Reflectance imaging spectroscopy is a hybrid technique that exploits two realities: spectroscopy and imaging. From the spectroscopy perspective, the ultraviolet, visible, and near-infrared light absorption characteristics of materials, as they are manifested from their diffuse reflectance features, characterize them. On the other hand, from the imaging perspective, MSI provides information from the spatial distribution of the different materials across a surface based on their reflection intensity. This unique combination enables mapping the materials’ spatial distribution across an illuminated surface. The unique abilities include the discrimination of materials having similar color appearance with different chemical structures. To achieve this, a large number of images from different wavelengths are combined, resulting in a full diffuse reflectance spectrum at every pixel (methodology based on homography techniques [29]). At the end of the pre-processing steps, all images compose the spectral cube, which is a three-dimensional space, represented by two dimensions for the surface information and one dimension for the wavelength. This is a rich dataset containing the chemical profile of the materials and their distribution across the surface. To understand the pigment distribution across the surface, advanced signal processing approaches are required to decompose the spectral cube and provide helpful information for the end user.

**Figure 4:**
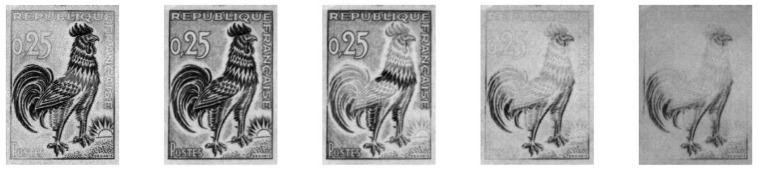
Multispectral images at 450 nm, 550 nm, 650 nm, 800 nm, and 1000 nm (from **left** to **right**).

In this work, the XpeCAM was pointed to gather a series of 23 images, each one corresponding to specific wavelength bands spanning from ultraviolet (370 nm) to infrared (1100 nm) (Figure 4 and Supplementary Materials, Video S1).

### 3.4. Co-Registration of μ-XRF and Multispectral Images

This section describes the procedure for pairing the μ-XRF recorded pixels to the MSI pixels, applying an algorithmic co-registration approach. This aligning process is essential in preparing the datasets for fusion [14]. To match the pixels, initially, it is necessary to bin the MSI pixels, of 30 × 30 μm^2^ size, to the same size as the μ-XRF pixels of 50 × 50 μm^2^. Integrating the SIFT (Scale-Invariant Feature Transform) algorithm enables the alignment of the images acquired through MSI to match the dimensions of XRF data. The well-known SIFT algorithm [30] is a sophisticated technique that comes from the computer vision sector for extracting and matching distinctive features from the multispectral image (set as source image) and the scanning μ-XRF image (set as target image), regardless of changes in scaling and lighting conditions. The algorithm begins by detecting critical points in both source and target images. This process is achieved by identifying areas with significant local contrast, such as corners, blobs, or edges. These key points serve as anchor points for the continuation of the process.

For creating the target image in question, elemental maps of Ca and Fe were super-positioned, creating an image resembling the RGB image. The deduced XRF image is compared to the 950 nm multispectral image in Supplementary Materials, Figure S1. After this process, key points were identified, and the algorithm generated descriptors for each. Once descriptors were generated, the SIFT algorithm established suitable matches between key points in the two images (source and target), as shown in Supplementary Materials, Figure S1. The matching process involves comparing the descriptors’ characteristics and finding correspondences that indicate the same feature in both images.

Finally, the transformation matrix is calculated to align points from one image to another. The RANSAC algorithm [31] was used to make the transformation robust against outliers in the correspondence data. This transformation matrix is applied to adjust the multispectral images to match the XRF target image’s perspective, orientation, and coordinates. This process was executed for all multispectral images. During co-registering MSI with XRF images, the scaling process was carefully investigated. In particular, the use of bilinear, cubic, and nearest neighbor interpolation techniques was investigated [32]. Notably, the results obtained from these different interpolation methods showed identical results. For this reason, bilinear interpolation was selected to be applied to the multispectral data.

## 4. μ-XRF and MSI Dataset Fusion for the Data Analysis

### 4.1. Composition Analysis Applying Multispectral Clustering and Mean XRF Spectra per Cluster

In multispectral data analysis, clustering techniques are crucial in uncovering patterns and relationships. The clustering of multispectral data was performed using the well-known k-means algorithm, chosen for its simplicity [33,34]. K-means is an iterative clustering algorithm that begins with randomly initializing cluster centers. Throughout each iteration, two vital steps are taken to refine the clusters. First, it assigns each spectrum to the cluster with the nearest centroid using a metric distance as a proximity measure. Then, it recalculates the centroids of these newly grouped clusters. This iterative process helps shape tightly packed clusters with minimal variation between them. Because k-means effectiveness can vary based on the initialization of the centroids, we executed the algorithm several times from different random initial partitions. In this manner, we attained a solution that minimizes the differences within groups while simultaneously maximizing the dissimilarities between them. This approach contributes to the generation of more coherent and reliable results.

The primary purpose of clustering here is to group similar MSI spectra. More specifically, the applied clustering method partitions the multispectral dataset into seven groups so that spectra in each cluster are close in terms of the Euclidean distance measure [33]. Since spectra in the same cluster are similar, the information in each cluster can be summarized by a representative spectrum, the cluster centroid (“endmember” spectra). By studying these centroid spectra, we can quickly grasp the spectral characteristics present in the dataset. Moreover, in the case of spectral images, the spatial distribution of the clusters across the image can be visualized by plotting an image where different parts are colored according to which cluster they belong to. This helps us visualize how clusters are distributed across the image, highlighting patterns and relationships in the data.

**Figure 5:**
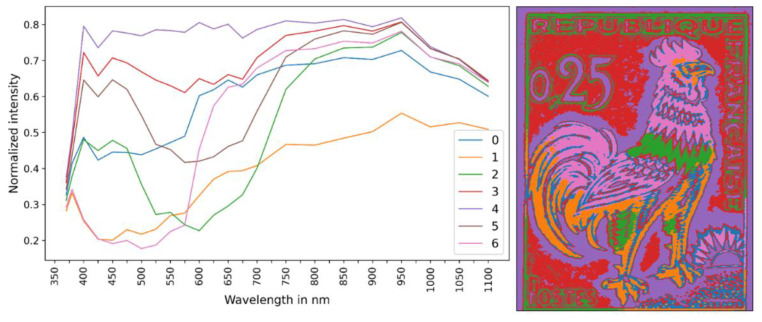
(**Left**) Endmember spectra of the MSI clusters (see text). (**Right**) The spatial distribution of the MSI clusters following the endmember distribution. The pixel size is equal to 50 × 50 μm^2^. The colors in the right image correspond to the cluster numbers listed in the left figure.

Figure 5 provides a comprehensive overview of the results achieved through the clustering analysis of multispectral data. The left part of the figure shows the endmembers of the clustering process applied to the MSI data, while the right part shows the spatial distribution of the seven different clusters. Each cluster is identified by a unique color, highlighting areas on the image characterized by similar spectral features/traits and allowing for insight into the spatial distribution of clusters across the image.

**Figure 6:**
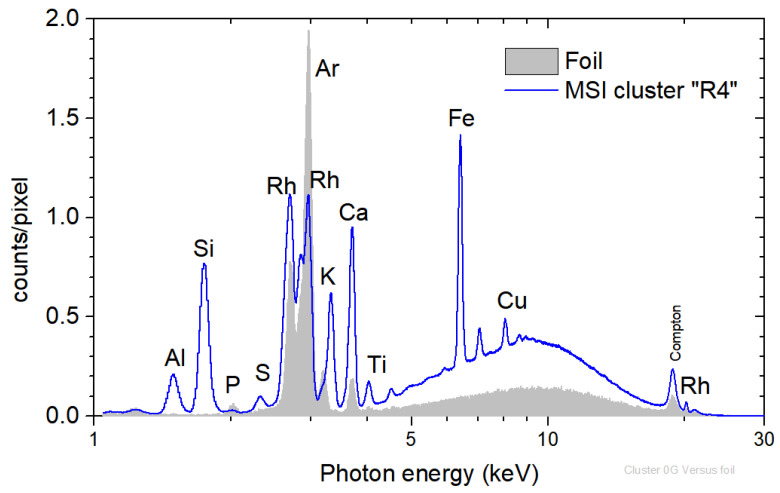
(**Left**) MSI cluster “R4” corresponding to the stamp’s unprinted paper; (**right**) mean XRF spectrum cluster “R4” compared to the mean XRF background spectrum (see text).

**Figure 7:**
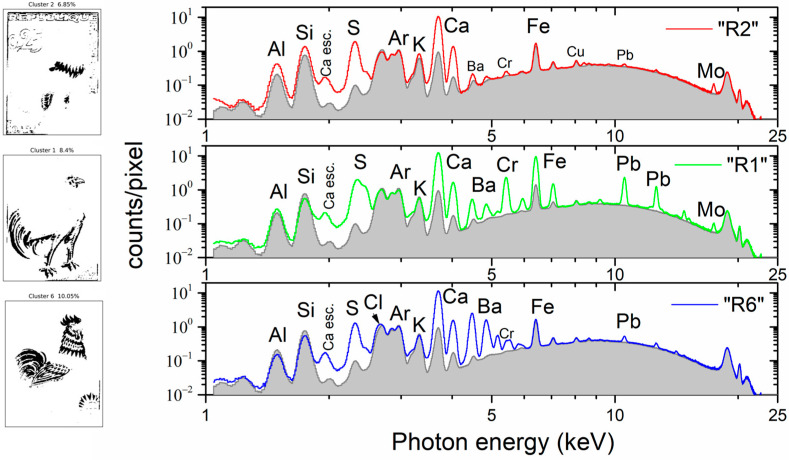
MSI clusters and corresponding mean XRF spectra. (**Top**) MSI cluster “R2” corresponds to the ultramarine blue area, (**middle**) MSI cluster “R1” corresponds to the lead chromate brown area, and (**bottom**) MSI cluster “R6” corresponds to the carmine red area. The spectra are compared to the mean XRF spectrum of the stamp paper (MSI cluster “R4”). Ca esc. indicates the escape peak of the Ca Κα transition. The arrow indicates the energy position of the Cl Κα.

The MSI cluster “R4” is closely linked to the white color of the unpainted paper substrate, as seen in Figure 5 and the stamp image in Figure 2. This is supported by the endmember spectrum, which exhibits constant high intensities throughout the entire wavelength range. Cluster “R4” covers 25.3% of the entire image. The corresponding mean XRF spectrum of cluster “R4” is shown in Figure 6 in comparison with the background spectrum. The X-ray fluorescence transitions of Al, Si, S, K, Ca, Ti, Fe, and Cu were observed. Rh K and L scattered transitions and Ar K transitions from the air were also detected. Notably, the observed Ca and Fe Κ XRF transitions, which imply the presence of Ca and Fe in the paper, are not identified on the transition XRF maps in Figure 3. This is because the Ca and Fe transition coming from pixels of the “R4” cluster have an intensity that is one order of magnitude lower than other areas of the stamp (see later in Figure 7). This makes it difficult to detect them due to the low contrast. It is important to remember that interpreting elemental maps requires careful attention. In an area where specific transition events are lacking, it may not necessarily mean their absence but rather that such events occur significantly less frequently than in other areas.

The MSI clusters named “R2”, “R3”, and “R5” are associated with the blue pigment present in the feathers on the rooster’s neck and legs, as well as in the letters on the stamp (Figure 5). Cluster “R2” corresponds to the most intensely blue-colored region of the stamp and covers 6.9% of the entire image, while “R3” and “R5” cover 26.4% and 11.1%, respectively. In these two clusters, we observe the characteristic shape of blue pigments, such as ultramarine and cobalt blue, with immersion in the MSI endmember between approximately 450 nm and 700 nm [35,36]. The mean XRF spectrum of cluster “R2” is shown in Figure 7 (top), compared to the stamp’s paper cluster, “R4”. The significantly enhanced intensity of the Al and Si K X-ray fluorescence transitions confirms the presence of ultramarine. Similarly, the significant increase in S and Ca K intensities, relative to the intensities in the stamp paper, indicates the presence of calcium sulfate filler (part of the S intensity increase should be attributed to the presence of ultramarine). The intensity of Fe and Cu remains constant, indicating that they originate from the stamp paper and are not attenuated by the blue pigment due to their high energy. The weak observed transitions of Ba, Cr, Pb, and Mo could originate from the pigment overlaps and/or the presence of traces (see Supplementary Materials, S3). The case of a deficient co-registration process was examined by re-evaluating the mean XRF spectrum, excluding all perimetric pixels of the cluster “R2”. Specifically, we retained only the pixels whose entire neighbors belong to the cluster. This reduced the number of pixels in the cluster “R2” considerably, and only 37% of the initial number of pixels was retained (see Supplementary Materials, S4). Confirmation of a successful co-registration process was achieved as no spectral differences were observed between the two mean XRF spectra.

The MSI cluster “R1” exhibits a clear association with the stamp’s darker regions, encompassing the brown hues found on the tail, legs, and head of the gallic cock, and it covers 8.4% of the entire image (Figure 7 (middle)). The mean XRF spectrum of cluster “R1” is shown in Figure 7, compared with the stamp’s paper cluster. The presence of Cr and Pb intense X-ray fluorescence transitions and the significant intensity enhancement of the Fe K transitions, relative to the intensity originating from the stamp paper, reveal the presence of lead chromate and iron ochre [27]. Moreover, the intensity of the S and Ca K transitions compared to the stamp paper indicates the presence of calcium sulfate filler. The Mo Kα transition has a similar intensity to the blue-colored region (MSI cluster “R3”). Finally, the Ba L transitions should be attributed to the overlap between brown and red pigments (see later discussion for cluster “R6”).

The MSI cluster “R6” corresponds to the vivid red regions adorning the cock’s comb, feathers, and rooster’s plumage on the stamp. It covers 10.1% of the entire image. The end-member spectrum of the MSI cluster “R6” exhibits a marked and rapid ascent, peaking at approximately 570 nm (Figure 5), corresponding to madder- and cochineal-based red pigments [37]. This result agrees perfectly with the literature reference that identifies the red pigment on this stamp as cochineal [26]. The mean XRF spectrum of “R6” is shown in Figure 7—bottom, relative to the stamp paper’s mean spectrum. The enhanced intensity of the S, Ca, and Ba X-ray fluorescence transition indicates the presence of calcium and barium sulfate. The low intensities of Cr and Pb transitions and the slight increase in Fe Kα are attributed to the overlap between the red and brown pigments.

### 4.2. Dataset Fusion for Comparing the Composition of “Similar” Stamps

**Figure 8:**
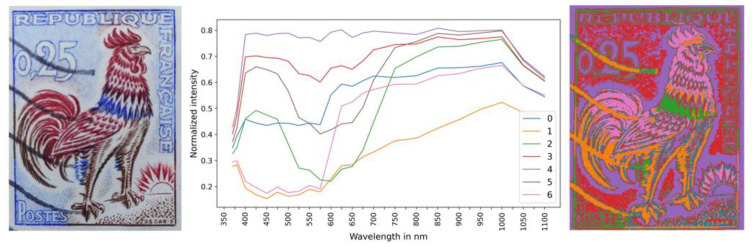
(**Left**) Stamp-B: 0.25 franc French Gallic cock stamp with postage postmark. (**Middle**) Endmember spectra for stamp-B. (**Right**) Spatial distribution of the MSI clusters following the endmember distribution. The pixel size is equal to 50 × 50 μm^2^.

The developed MSI and μ-XRF datasets’ fusion analysis was applied to compare the composition of two “similar” 0.25 franc French Gallic cock stamps, namely stamp-A and stamp-B. Stamp-A is the stamp examined thus far (Figure 2). In contrast, stamp-B is shown in Figure 8 (left). Stamp-B has been measured by MSI and scanning μ-XRF under identical experimental conditions as stamp-A (Supplementary Materials, Figure S6). Stamp-B features a postage postmark consisting of four stripes.

The endmember spectra of stamp-B are shown in Figure 8 (middle) (XRF elemental distribution maps are given in Supplementary Materials, Figure S7). The MSI clusters distribution of stamp-B (Figure 8 (right)) exhibits a notable similarity with the MSI clusters of stamp-A (Figure 5 (right)), which was anticipated as the spatial chromatic distribution is comparable for both stamps. The MSI clustering and corresponding mean XRF spectrum per cluster enable direct comparison of equivalent areas in the two stamps.

**Figure 9:**
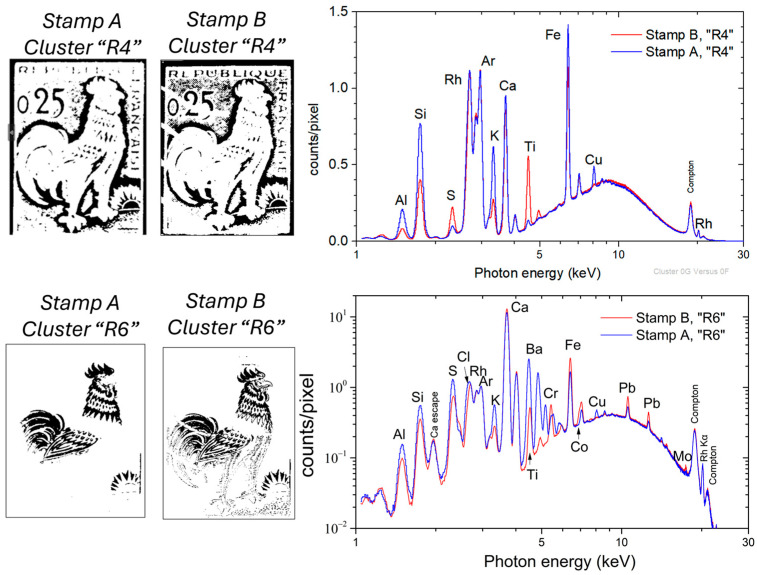
(**Top**) MSI cluster “R4”, the unprinted areas for stamp paper A and B, and the corresponding mean XRF spectra. (**Bottom**) MSI cluster “R6”, the red-printed areas for stamp paper A and B, and the corresponding mean XRF spectra. The arrows indicate the energy positions of Κα transitions of the corresponding elements.

The paper composition of the stamps is compared by examining the mean XRF spectra of the MSI clusters, “R4”, associated with the white area of the stamps (Figure 9 (top)). The mean XRF spectra of both “R4” clusters are shown in Figure 9. The dominant intensities correspond to Ca and Fe transitions in both stamps. The scattered Rh and Ar transitions show negligible variations. The intensity of Al, Si, and K transitions is lower in stamp-B than in stamp-A, while the Ti and S intensities are higher in stamp-B. The Cu K transition is not observed in stamp-B. Based on the extracted result, it is concluded that the two stamps were printed on different types of paper.

The mean XRF spectra of the stamps’ red-colored areas, corresponding to the MSI cluster “R6”, are shown in Figure 9 (bottom). Stamp-A shows a strong presence of barium, while stamp-B does not. It is worth noting the detection of Co in stamp-B (see Supplementary Materials, Figure S5). The higher intensities of Cr, Fe, and Pb transitions in stamp-B indicate greater overlap between the red and brown pigments than in stamp-A. The differences in elemental composition between the two stamps show variations in pigment, providing insights into material characteristics and potential differences in printing processes.

### 4.3. Composition Analysis by Sub-Clustering the μ-XRF Dataset within an MSI Cluster

**Figure 10:**
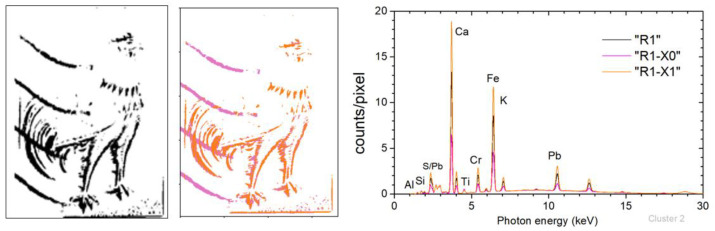
Stamp-B: (**left**) MSI cluster “R1” corresponding to the stamp’s brown area and postage postmark. (**Middle**) Spatial distribution of the XRF sub-clusters “R1-X0” (magenta) and “R1-X1” (orange). (**Right**) Mean XRF spectra of the MSI “R1” cluster and the associated XRF sub-clusters (see text).

MSI cluster “R1” in stamp-B incorporates regions occupied by the brown pigment and the black seal (Figure 10 (left)). During the MSI clustering process, black and brown pixels were merged into the same cluster as they exhibited reduced intensity reflection spectra. It is evident that while these pixels exhibit similar behavior in MSI, their XRF spectra must differ significantly. The seal’s four black strips lie either on the blue (ultramarine) pixels or on the brown (lead chromate and iron) pixels.

A sub-clustering based on the XRF spectra of the MSI “R1” pixels was applied, to differentiate the pixels according to their elemental composition. This is justified as the XRF clustering is based on the intensity strength of the characteristic fluorescent lines in each spectrum [38,39], which reflects the elemental composition. Specifically, the k-means method is applied for the XRF sub-clustering, with k = 2. The application of XRF sub-clustering results in two well-differentiated clusters, given in Figure 10 (middle). The cluster “R1-X1” is well associated with the brown area of the stamp, while the cluster “R1-X0” corresponds to the postage postmark, specifically the black part of the stamp. The mean XRF spectra of the two XRF sub-clusters are shown in Figure 10 (right). The non-zero intensity of the characteristic transitions of Pb, Cr, and Fe in the “R1-X0” sub-cluster originates from the overlapping regions between the postage stamp and the brown pigment. This is especially noticeable in the third strip (from the top) as it merges with the poultry tail.

### 4.4. Elemental Composition of the Postage Postmark

**Figure 11:**
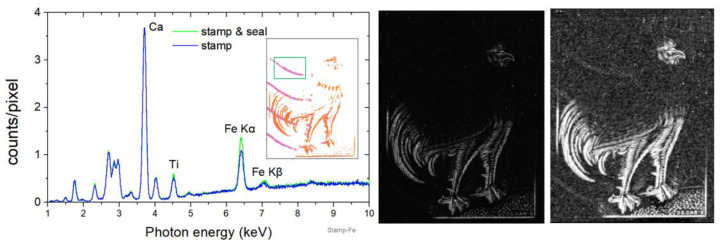
Stamp-B. (**Left**) The mean XRF spectrum of the seal strip at the top-left corner is compared with the XRF spectrum of the surrounding structure (see text). (**Middle**) The Fe Κα elemental map in linear intensity scale. (**Right**) The Fe Κα elemental map in logarithmic intensity scale.

The aim of this section is to extract information about the elemental composition of the postmark on stamp-B by examining the corresponding XRF spectrum. Knowledge of the postmark’s composition may be decisive for extracting information about the postmark’s origin. It is essential to detach the seal from the adjoining structure to accomplish this task. Moreover, the X-ray fluorescence spectrum of the surrounding structure needs to be utilized to determine the substrate’s contribution to the seal’s measured fluorescence spectrum. One of the four seal strips was separated from its surrounding structure at the top-left corner of the stamp in MSI/ XRF sub-cluster “R1-X0” (strip within the green square in the inset of Figure 11 (left)). The seal’s mean XRF spectrum was extracted by selecting only the pixels within the green box of sub-cluster “R1-X0” (Figure 11 (left)). However, this spectrum contains a spectral contribution originating not only from the seal’s elemental composition but also from the underlying structure. To determine the spectral contribution of the underlying structure, we evaluated the mean XRF spectrum for a structure identical to the seal shifted by twenty pixels upwards. The two spectra are compared in Figure 11 (left). An increase in Fe K transitions’ intensity can be observed, while the rest of the fluorescence spectrum remains unaffected. This reveals the presence of iron in the sealed ink. It should be noted that, upon examination of the Fe Kα intensity map, no iron imprint was detected within the postmark’s area (Figure 11-middle). This is due to the contrast between low-intensity iron in the seal and high intensity in brown and red pigments. However, visualizing the Fe Kα intensity map using a logarithmic intensity scale, the iron on the seal is visible (Figure 11 (right)). It should be emphasized that the ability to collect the pixels of the stamp significantly improves the spectrum statistics and offers the possibility of quantitative analysis.

## 5. Conclusions

In the present study, a complex-in-structure stamp, the 0.25 franc French Gallic cock stamp created by Albert Decaris, was analyzed using multimodal imaging and spectroscopic techniques such as scanning μ-XRF and MSI, with a high spatial resolution and a pixel size of a few tens of micrometers in both directions. The individual datasets were acquired asynchronously. The study is based on the fusion of μ-XRF and multispectral datasets. The dataset fusion was achieved through two steps: first, co-registration, and then clustering and sub-clustering between the multispectral and μ-XRF datasets.

The co-registration algorithm aligns MSI and XRF images and bins the smaller MSI pixel size to match the μ-XRF pixel size. The pixel sizes are as small as a few tens of micrometers. Multispectral clustering was deduced by applying the k-means clustering on the acquired MSI dataset. The determination of the MSI cluster originates from the wavelength-dependent light reflection from the sample’s surface based on color differentiation and/or pigment distribution. Subsequent XRF k-means sub-clustering within an MSI-defined cluster enables the identification of areas with diverse elemental compositions but similar light reflectance properties. This is justified as the XRF clustering is based on the intensity strength of the characteristic fluorescent lines in each spectrum. The validity of the data fusion approach was confirmed by examining the mean XRF corresponding to the MSI clusters of the measured stamp. The mean XRF spectrum of each cluster showed intense X-ray fluorescence transitions, corresponding to the expected main elements based on the pigment compositions of the stamp. To the best of our knowledge, this is the first attempt at implementing XRF sub-clustering within an MSI cluster.

The fusion of datasets was applied to compare specific regions of two 0.25 franc French Gallic cock stamps, to extract information about the stamps’ paper, to isolate specific regions, and to identify the elemental composition in the postage postmark. The presence of ultramarine, cochineal, and lead chromate with iron ochre is verified. The dataset fusion considerably enhances the analytical capability of the two spectroscopic imaging techniques, as the pixels’ spectral analysis within clusters may provide significantly more information than analyzing the same pixels as part of the entire dataset.

## Data Availability

The authors confirm that the data supporting the findings of this study are available within the article and its Supplementary Materials.

## Supplementary Materials

The following supporting information can be downloaded at https://www.mdpi.com/article/10.3390/jimaging10040095/s1, Figure S1: Matching Process; Figure S2: Mean XRF Spectrum per Multispectral Cluster; Figure S3: Pigment Overlap; Figure S4: Co-registration process; Figure S5: Mean XRF spectra of stamp-A and stamp-B; Figure S6: Elemental distribution maps of stamp-B; Figure S7: MSI cluster mapping of stamp-B; Video S1: MSI images.
