# Supplementary material for: Scanning Micro X-ray Fluorescence and Multispectral Imaging Fusion: A Case Study on Postage Stamps
Source: J Imaging. 2024 Apr 22;10(4):95. doi: 10.3390/jimaging10040095 (PMC11051289; doi:10.3390/jimaging10040095)
Supplement: Supplementary file 1 [file jimaging-10-00095-s001.zip › jimaging-2935404-supplementary v.02.pdf]

Supplementary material

S1. Matching Process

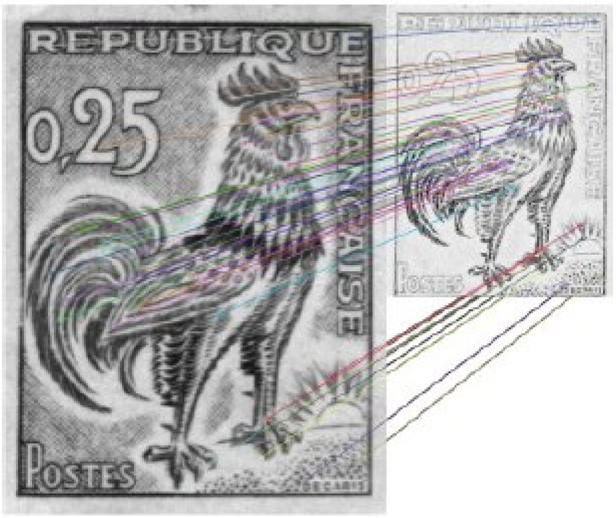

Figure S1. During the matching process, common features for the MSI 950 nm image (left) and the Ca and Fe Kα micro-XRF transition maps (right).

S2. Mean XRF Spectrum per Multispectral Cluster

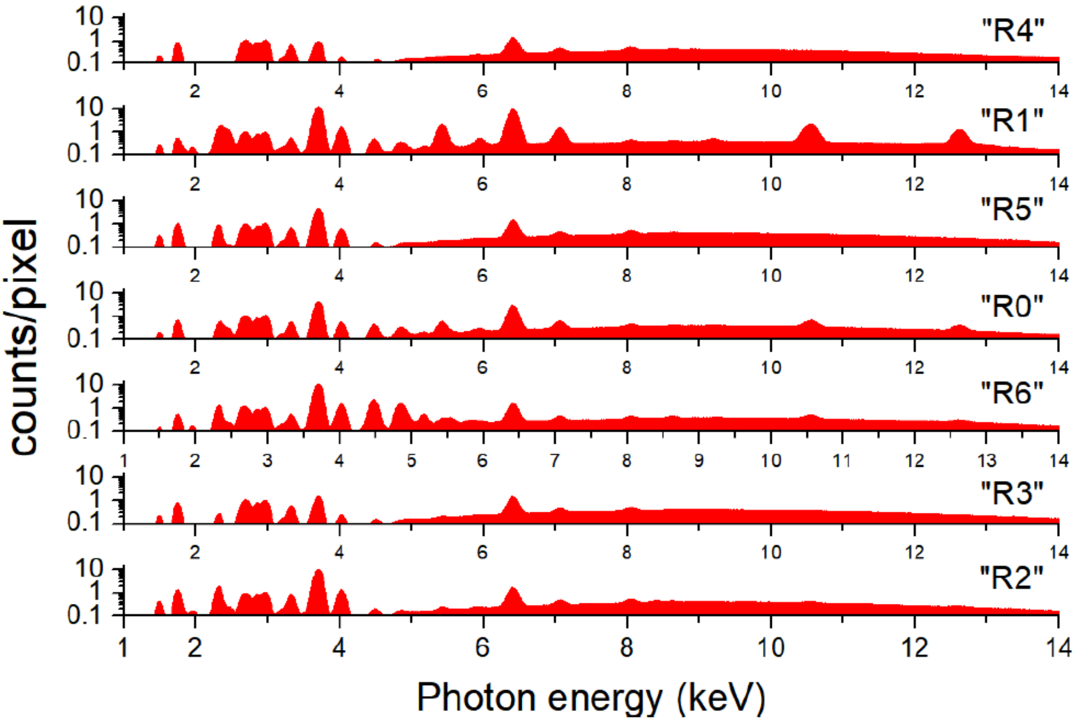

Figure S2. Mean XRF spectra for each of the MSI clusters as shown in Figure 5 (right). More detailed analysis for clusters "R4", "R2", "R1", and "R6" is given in the paper.

S3. Pigment Overlap

To assess the method's ability to detect trace elements within the MSI cluster "R4" (blue area) of stamp-A, the cluster spectrum was divided into three subareas based on their proximity to other pigments (Figure S3). Comparing these subareas' spectra with the mean XRF spectrum of the entire "R4" cluster revealed elements associated with the

cluster itself (e.g., Cu, Si, Al) and those influenced by neighboring pigments (e.g., Ba in subarea 2 due to the red dye). Subarea 1, lacking adjacent pigments, as shown in Figure 2, displayed a smoother spectrum without neighboring element traces, suggesting potential issues with co-registration or overlapping boundaries.

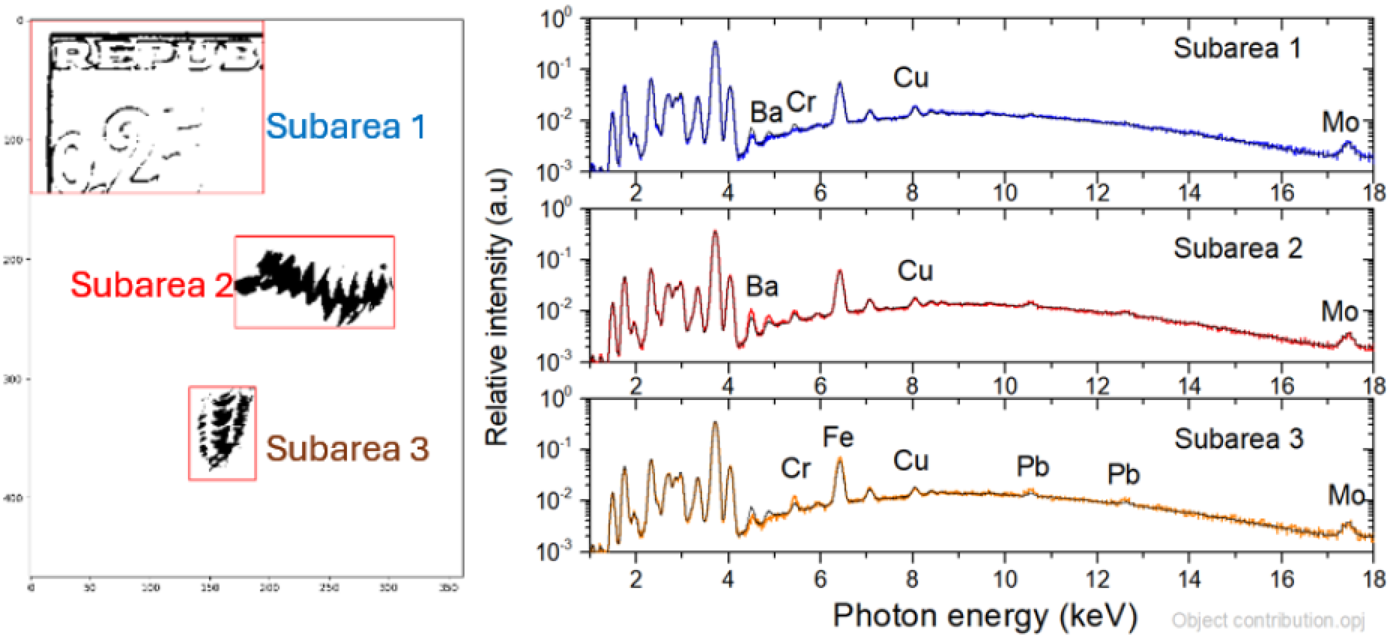

Figure S3. (Left) Subareas 1, 2, and 3 were selected to compare the XRF mean spectra with the overall mean XRF of the entire blue area of the stamp (MSI cluster "R2"). (Right) Plots of XRF mean spectra for the three selected areas, compared with the mean spectra of the entire blue area.

S4. Co-registration process

A comprehensive analysis was conducted to investigate a potential deficiency in the co-registration process by re-evaluating the mean XRF spectrum with a focused approach. This re-evaluation excluded all perimetric pixels associated with the cluster "R2". To ensure precision, only pixels with entirely neighboring pixels belonging to the cluster were retained for further analysis (see Figure S4). This meticulous refinement significantly reduced the number of pixels within cluster "R2", resulting in a retention rate of only 37% compared to the initial count (see Table S4). This approach confirmed the presence of targeted elements (Cr, Ba, Cu, Pb) but highlights the need for caution when interpreting trace element data due to potential limitations like co-registration and overlapping pigments.

Table S1. Percentage (%) of pixels from the initial MSI clusters of stamp-A that remain after cropping.

RIS cluster	% of pixels
R0	12
R1	52
R2	37
R3	39
R4	69
R5	20
R6	55

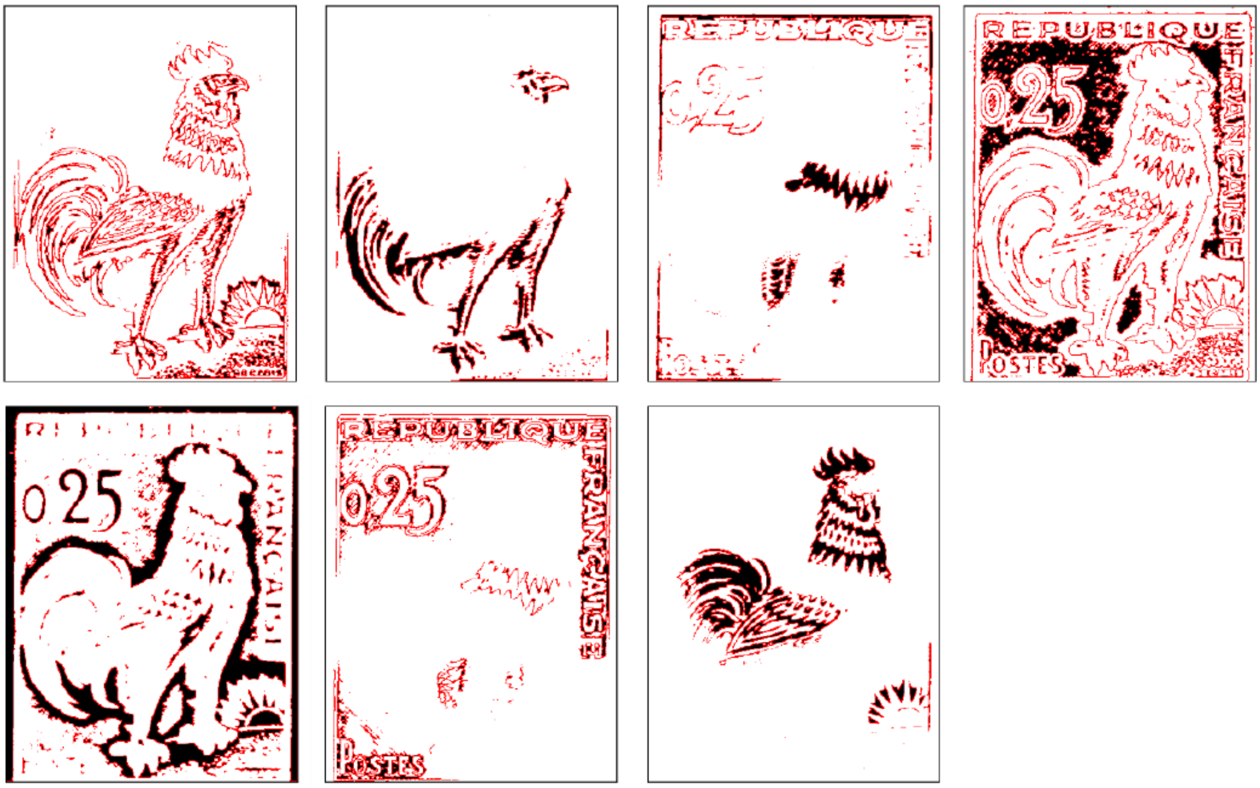

Figure S4. (Upper from left to right) MSI cluster masks of stamp-A from 0 to 4. (Bottom from left to right) MSI cluster masks of stamp-A from 5 to 7. The plot illustrates the delineation of perimetric pixels of each MSI cluster. Red denotes the exclusion of perimetric pixels, while black outlines the retained pixels within the clusters after cropping.

S5. XRF mean spectra, elemental distribution maps, and MSI cluster mapping

In Figure S5, each spectrum represents the average elemental composition of each stamp, providing insights into the distribution of various elements present. Additionally, Figure S6 includes elemental distribution maps of stamp-B, providing visual insights into the spatial distribution of elements across the stamp's surface. Finally, in Figure S7, we observe MSI cluster mapping of stamp-B.

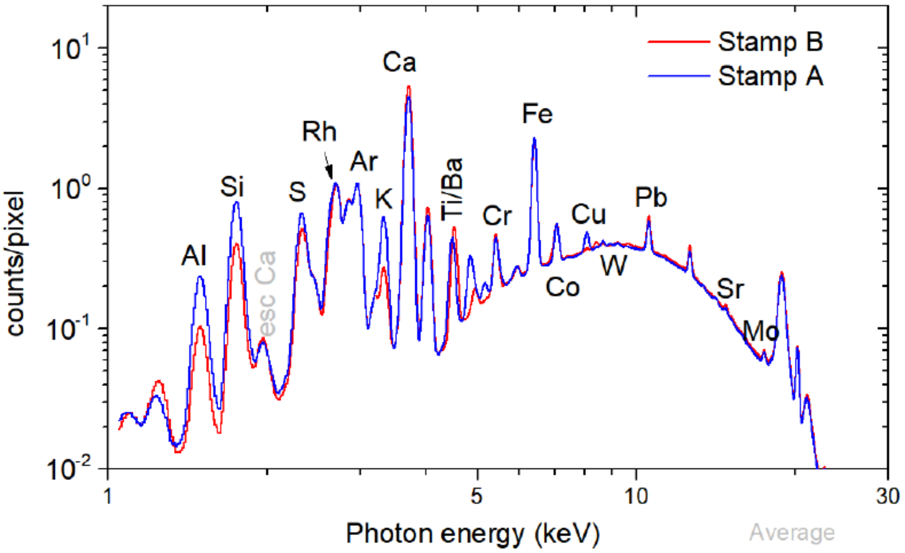

Figure S5. Mean XRF spectra of stamp-A and stamp-B.

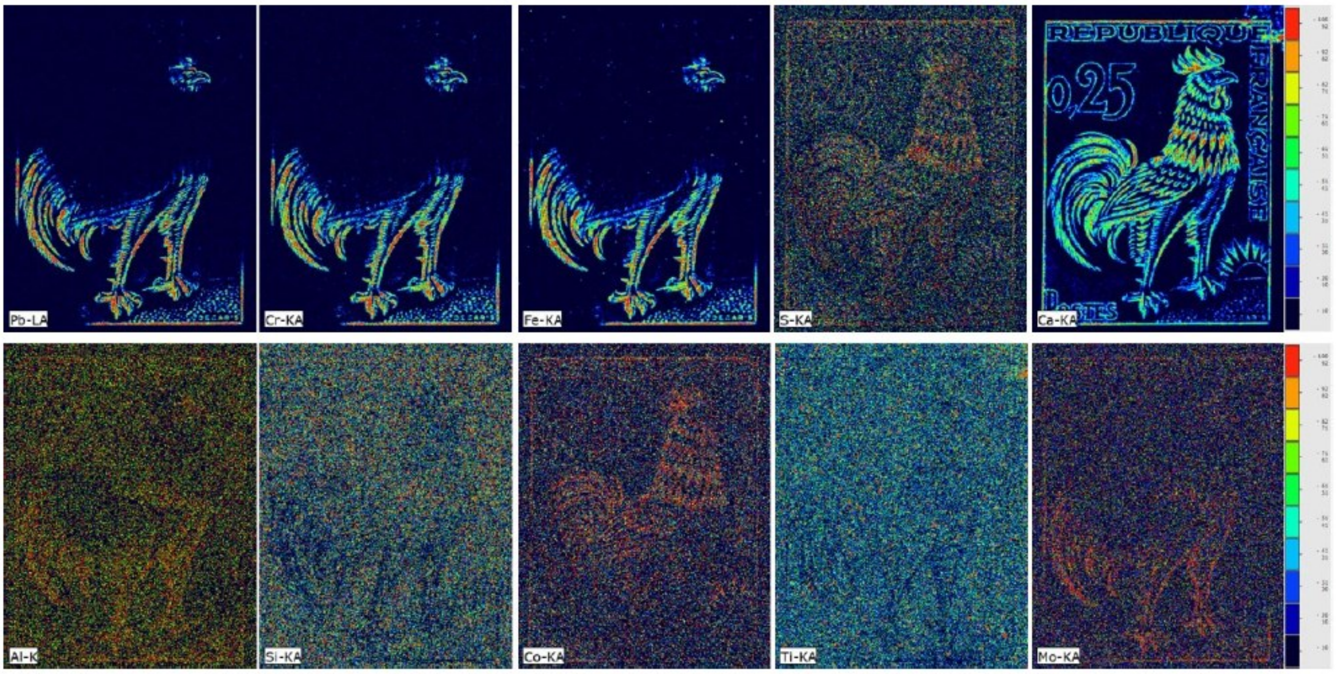

Figure S6. Elemental distribution maps of stamp-B.

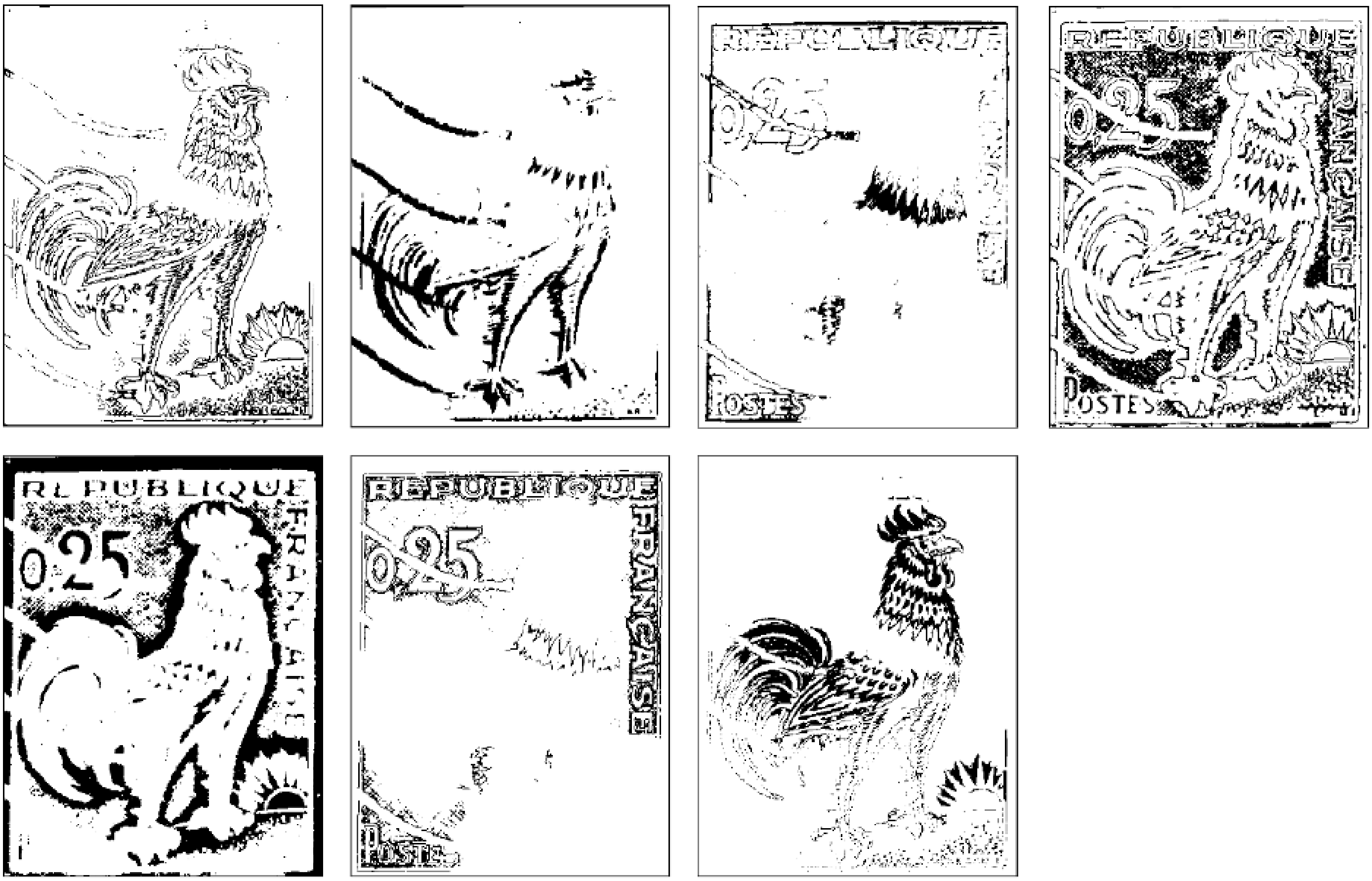

Figure S7. MSI cluster mapping of stamp-B. (Upper from left to right) From “R0” to “R4”. (Bottom from left to right) From “R5” to “R7”.
